# Editorial: Advances in Invasive and Non-invasive Brain Stimulation in Parkinson's Disease: From Basic Science to New Technologies

**DOI:** 10.3389/fneur.2022.914102

**Published:** 2022-06-20

**Authors:** Maria Sheila Guimarães Rocha, Camila Catherine Aquino, Marina Picillo, Rubens Gisbert Cury, Fabio Godinho

**Affiliations:** ^1^Hospital Santa Marcelina, São Paulo, Brazil; ^2^Neurology Department, Santa Marcelina College, São Paulo, Brazil; ^3^Neurology Department, University of Calgary, Calgary, AB, Canada; ^4^Neurology Department, University of Salerno, Fisciano, Italy; ^5^Department of Functional Neurosurgery, University of São Paulo, São Paulo, Brazil

**Keywords:** Parkinson's disease, brain stimulation, local field potential (LFP), transcranial direct current stimulation (tDCS), repetitive transcranial magnetic stimulation (rTMS), sleep disturbance, deep brain stimulation (DBS)

Parkinson's disease (PD) is the second most prevalent neurodegenerative disease, substantially impacting quality of life and economic burden. Currently available treatments, including pharmacological interventions, rehabilitation, and brain stimulation, undoubtedly help to reduce disease symptoms. This Frontiers of Neurology unique Research Topic brings us several new insights into improving brain stimulation interventions.

Brain stimulation is one of the fastest-growing neuroscience areas involving medical and bioengineering fields. Brain stimulation is inherently non-destructive, reversible, and, most importantly, adjustable. Whether invasive or non-invasive, the electrical intervention can modulate the nervous system function, leading to improved neurological symptoms and better quality of life.

Deep brain stimulation (DBS) has been clinically useful in the treatment of PD at all stages, especially in those patients with motor symptoms only partly controlled by dopaminergic drugs, such as severe rest tremor or off-period dystonia, and motor fluctuations. However, many DBS issues remain challenging, for instance, choosing a suitable stimulation target to maximize clinical outcomes, while minimizing side effects. As a highly heterogeneous disease, one DBS solution does not fit all patients.

Neurosurgeons commonly face the challenge of precisely localizing tiny surgical targets. Indeed, successful application of DBS relies on optimal lead placement, among several factors. In this regard, Shi et al. used microstimulation during microelectrode recordings to localize the subthalamic-substantia nigra border. The authors provided evidences that it can be easily and routinely employed to achieve better lead placement in the STN and superior therapeutic effectiveness.

There has been much debate on the best DBS target for PD. DBS targeting the subthalamic nucleus (STN) and the internal Globus pallidus (Gpi) reduces PD's motor and non-motor symptoms. Comparative studies suggested that STN and GPi DBS have similar outcomes. Nevertheless, GPi DBS likely causes less impact on both gait and cognition. Zeng et al. assessed the outcome differences of stimulating STN or GPi in the same individual. A significant improvement in motor symptoms occurred after STN stimulation. Effects of unilateral STN stimulation were seen on both sides of the body, while unilateral GPi stimulation mainly acted on the contralateral side, thus providing evidence favoring STN DBS.

Adaptive DBS has gained space in the research and clinical fields. This novel approach might improve troublesome side-effects from conventional DBS, such as speech disturbances, disabling gait disorders, and behavioral changes. By enabling the recording of patient-specific local field potential (LFP) signals through electrode contacts adjacent to the stimulating electrode contact of the same DBS lead, adaptive DBS may allow for automated brain stimulation adjustment and better management of PD symptoms. This closed-loop approach demands familiarity with electrophysiological biomarkers associated with distinct clinical manifestations. On this field, Baumgartner et al. gifted us with a comprehensive review of the relationship between LFP oscillations in the STN and the sleep architecture of PD patients. This knowledge may allow future closed-loop optimization of electrical parameters to treat sleep dysfunction in PD.

Many factors may contribute to DBS outcomes in PD, and the genetic profile is undoubtedly one of them. About 25% of individuals undergoing DBS have a genetic form of PD. Given the individual variability in clinical evolution and surgical responses, it is reasonable to hypothesize that genetic variability may relate to distinct phenotypes and DBS outcomes. Accordingly, patients with LRRK2, parkin, VPS35, and SNCA mutations respond well to DBS treatment, whereas patients with glucocerebrosidase (GBA) mutations may disclose faster cognitive decline and poorer responses following DBS.

Beyond neuropathological issues, electrophysiological differences may justify differences in DBS outcome. David et al. analyzed the differences between left and right STN resting-state beta power in GBA mutation carriers with PD. The differences in peak beta ratio in GBA-mutation carriers correlated to the clinical findings, suggesting a distinctive physiologic signature from sporadic PD. Additional research on LFP attributes according to the PD genetic profile will provide resources for adaptive DBS programming.

DBS research may also explore alternative ways of electrical stimulation. An animal study by Wang et al. demonstrated the impact of coordinated STN DBS reset on motor parkinsonism. Preliminary evidence supports shuffled STN CR-DBS producing significantly better therapeutic effects on parkinsonian symptoms, with the additional gain of reducing side-effects by minimizing the current spread.

The systematic review, by Miao et al. on functional magnetic resonance imaging (fMRI) to investigate modulatory DBS effects on brain activity, shed light on DBS impact on functional connectivity. The authors reviewed studies on the mechanism of DBS action, the effects of chronic stimulation on motor networks, the impact on different inter-regional connectivity, the effects on non-motor symptoms in PD, and differences in levodopa and DBS actions on brain activity.

DBS immediately modulates the cortico-basal ganglia-thalamocortical loop, leading to significant physiological modifications in the thalamus, globus pallidum, and cerebellum. The primary motor cortex activation changes correlate with motor symptoms and PD phenotypes. The impact of DBS on brain activity depends on several factors, such as programming parameters, subject's activity while being scanned, PD subtypes, and medication intake. Future use of fMRI should allow individualized surgical planning and help identify optimal anatomical targets as per symptoms. Overall, fMRI must enhance the understanding of DBS mechanisms in PD and help to improve clinical outcomes.

Non-invasive brain stimulation (NIBS), as theta-burst stimulation (TBS), proposes managing several PD symptoms. Furthermore, novel targets for rTMS, such as the prefrontal cortex, motor cortex, cerebellum, and spinal cord, focus on different symptoms like depression, apathy, motor symptoms, and gait disturbance. The mini-review performed by Wu et al. broadly discusses the role of rTMS and TBS in LID in PD. The authors explored the therapeutic mechanism of TMS in the management of LID, which involves understanding many neural circuits that take part in the occurrence of LID. Identifying brain regions involved in LID mechanisms is critical. The right stimulation target or combination of different areas might prolong therapeutic efficacy. Pan et al. highlighted the shortness of TMS efficacy protocols. They showed that high-frequency rTMS over the left dorsolateral-prefrontal cortex (DLPFC) only provides short-term improvements for alleviating fatigue in patients with multiple system atrophy.

Cheng et al. systematically and quantitatively analyzed the therapeutic effect of TBS for PD treatment. TBS leverages repetitive TMS due to its short time of single treatment and low stimulation intensity compared to traditional rTMS. Accordingly, TBS over the supplementary motor area significantly improved motor burden in the off-medication period. Additionally, intermittent TBS over the motor cortex and DLPFC impacted the slowing of gait and depression.

NIBS may change quantitative electrophysiological signs in PD patients. The scoping review of Costa et al. gathered evidence of the neurophysiological changes associated with NIBS in PD. They evidenced the NIBS' impact on the cortical activity as measured by electroencephalogram. On the other hand, the systematic review by Oliveira et al. found no significant short-term effect of tDCS on motor function, balance, gait, dyskinesia, or motor fluctuations in PD, regardless of brain area or targets stimulated. These opposite findings might reflect differences in the quality of the studies, the low number of studies, and especially variability in NIBS intervention.

NBIS new technologies, such as galvanic vestibular stimulation (GVS), are being increasingly explored in PD. Lee et al. investigated the behavioral GVS effects under different stimulation frequencies and the interaction between GVS effects and anti-parkinsonian medication. Clinical response varied considerably across participants under the tested conditions. Moreover, dopaminergic drugs significantly influenced GVS effects in PD patients. Kazemi et al. searched for EEG predictive measures of impaired motor vigor in PD, which may provide valuable leads for GVS modulation.

Finally, Pfeifer et al. shared the study protocol on clinical efficacy and dosing of vibrotactile coordinated reset stimulation (VCR) in PD symptoms. VCR is a non-invasive therapy that delivers gentle vibrations to the fingertips. VCR might desynchronize abnormal brain rhythms within the sensorimotor cortex, thus relieving motor and non-motor symptomatology in PD.

From the evidence shown in this Research Topic, advances in brain stimulation are encouraging, but there are still many critical issues to address. We must fully clarify its mechanisms of action at the cellular level, its related neurophysiological events, and its impact on the regular and pathological neuronal networks. Besides, it is imperative to use multimodalities of PD biomarkers to better predict the outcome at the individual level as a tool for individualized medicine. Integrating neurophysiology, neuroimaging, and genomics into patient care is a highly strategic priority.

## Author Contributions

All authors listed have made a substantial, direct, and intellectual contribution to the work and approved it for publication.

## Conflict of Interest

The authors declare that the research was conducted in the absence of any commercial or financial relationships that could be construed as a potential conflict of interest.

## Publisher's Note

All claims expressed in this article are solely those of the authors and do not necessarily represent those of their affiliated organizations, or those of the publisher, the editors and the reviewers. Any product that may be evaluated in this article, or claim that may be made by its manufacturer, is not guaranteed or endorsed by the publisher.

